# Low-volume muscular endurance and strength training during 3-week forearm immobilization was effective in preventing functional deterioration

**DOI:** 10.1186/1476-5918-7-1

**Published:** 2008-01-15

**Authors:** Mika Matsumura, Chihoko Ueda, Kiyoshi Shiroishi, Kazuki Esaki, Fumiko Ohmori, Kuniko Yamaguchi, Shiro Ichimura, Yuko Kurosawa, Ryotaro Kime, Takuya Osada, Norio Murase, Toshihito Katsumura, Akinori Hoshika, Takafumi Hamaoka

**Affiliations:** 1 Department of Sports Medicine for Health Promotion, Tokyo Medical University, 6-1-1 Shinjuku, Shinjuku-ku, Tokyo, 160-8402, Japan; 2 Department of Pediatrics, Tokyo Medical University, 6-7-1, Nishi-Shinjuku, Shinjuku-ku, Tokyo, 160-0023, Japan; 3 Research Institute of Physical Fitness, Japan Women's College of Physical Education, 8-19-1 Kita-Karasuyama, Setagaya-ku, Tokyo, 157-8565, Japan; 4 Department of Judotherapy and Sports Medicine, Faculty of Health Science, Ryotokuji University, 5-8-1 Akemi, Urayasu-City, Chiba, 279-8567, Japan; 5 Department of Sports Medicine and Science, National Institute of Fitness and Sports in Kanoya, Shiromizu-cho 1, Kanoya, Kagoshima, 891-2393, Japan; 6 Department of Food and Nutrition, Yamanashi Gakuin Junior College, 2-4-5 Sakaori, Kofu City, Yamanashi, 400-8575, Japan; 7 Faculty of Science and Technology, Tokyo University of Science, 2641 Yamazaki Noda City, Chiba, 278-8510, Japan; 8 Department of Neurology, University of Cincinnati, 3125 Eden Ave, 2327 Vontz Center for Molecular Studies PO Box 670536 Cincinnati, OH, 45267-0536, USA

## Abstract

**Purpose:**

The purpose of this study was to determine whether endurance and strength hand grip exercises during 3-week upper limb immobilization preserve muscle oxidative capacity, endurance performance and strength.

**Methods:**

Ten healthy adult men underwent non-dominant forearm immobilization by plaster cast for 21 days. Five healthy adult subjects were designated as the immobilization (IMM) group and five were designated as the immobilization + training (IMM+TRN) group. Grip strength, forearm circumference, dynamic handgrip endurance and muscle oxygenation response were measured before and after the 21 day immobilization period. Using near-infrared spectroscopy (NIRS), muscle oxygen consumption recovery (VO_2_mus) was recorded after a submaximal exercise and the recovery time constant (TcVO_2_mus) was calculated. Reactive hyperemic oxygenation recovery was evaluated after 5 minutes ischemia. Two training programs were performed by the IMM+TRN group twice a week. One exercise involved a handgrip exercise at 30% maximum voluntary contraction (MVC) at a rate of 1 repetition per 1 second until exhaustion (about 60 seconds). The other involved a handgrip exercise at 70% MVC for 2 seconds with a 2 second rest interval, repeated 10 times (40 seconds).

**Results:**

There was a significant group-by-time interaction between the IMM and IMM+TRN groups in the TcVO_2_mus (p = 0.032, F = 6.711). A significant group-by-time interaction was observed between the IMM and IMM+TRN groups in the MVC (p = 0.001, F = 30.415) and in grip endurance (p = 0.014, F = 9.791). No significant group-by-time interaction was seen in forearm circumference and reactive hyperemic oxygenation response either in IMM or IMM+TRN group.

**Conclusion:**

The training programs during immobilization period used in this experiment were effective in preventing a decline in muscle oxidative function, endurance and strength.

## Background

Physical activity declines with aging and illness (particularly long-term hospitalization), and inactivity decreases various functions of varying organs. In particular, reduction of bone mineral density [1] and atrophy of skeletal muscle [2] are known to be results of disuse. There are also several reports on the influence of disuse on the blood vessel system measured by reactive hyperemic blood flow. For example, reactive hyperemic blood flow in the superficial femoral artery decreased by 28% after 52 days of bed rest [3]. Although not significant, a trend towards a decrease in hyperemic flow was present after 4 weeks of unilateral lower limb suspension [4]. These reports suggest that hyperemic blood flow, which is a marker for vasodilator capacity of resistance vessel, may be decreased after periods of inactivity.

Various models have been chosen depending on the purpose of the study, such as bed rest [5], natural aging [6], space flight [2,7], leg suspension [4,8], excision of nerve, leg immobilization [9] and forearm immobilization [10,11]. In this study, we have chosen the forearm immobilization model. The merits of this model are minimum restriction of daily life and less burden on subjects.

In contrast to conventional invasive methods [12] that present a great burden to patients, the use of a simple and non-invasive near infrared spectroscopy (NIRS) to monitor muscle oxidative metabolic function has come under attention [13-17]. NIRS is comparatively low in cost and also small and portable, so it can be used widely in clinical application [18-20]. In this study, we utilized NIRS for noninvasively evaluating muscle oxidative capacity and endothelial function.

By using a forearm immobilization model that can bring about muscle functional decline with specificity, we confirmed the deterioration of muscle oxidative capacity (-18% to -45%), endurance performance (-19.2% to -19.5%) and muscle strength (-17.9% to -15.6%) [10,11]. To preserve muscle functions, we performed low-volume endurance training (30%MVC, 1 contraction/second until exhaustion, twice a week) [11]. Although we were able to prevent decline of endurance and oxidative capacity by the endurance training, we were unable to prevent decrease in grip strength [11]. Previous studies [21,22], demonstrating that combination of strength and endurance training has preserved functional capacity during immobilization, motivated us to test the effect of combined exercise training on the forearm functions.

The purpose of this study was to determine whether endurance and strength hand grip exercises during 3-week upper limb immobilization preserve muscle oxidative capacity, endurance performance, as well as if strength and endothelial function decrease. In this study, we hypothesized that the decrease in the muscle strength would be reduced if the subjects performed strength training in addition to the endurance training during immobilization periods. Furthermore, we hypothesized that the 3-week forearm immobilization would decrease the oxygen delivery, while the exercise training may preserve the function.

## Methods

### Subjects

Ten healthy men participated in this study. The experiment was administered after undergoing a review by the National Space Development Agency of Japan (NASDA) ethics committee and receiving informed consent from the subjects. For all subjects, the non-dominant arm was immobilized for 21 days with a cast. The cast was placed from the fingers to the point two-thirds up the upper arm in the natural position. Subjects were instructed to wear slings in the daytime, except when changing clothes and bathing. Furthermore, they were unable to remove the casts by themselves during the study. Each subject was placed into one of two groups randomly, one group to undergo immobilization only (IMM, 5 subjects) and one group to undergo immobilization + training (IMM+TRN, 5 subjects). Measurements were made over 2 days for both IMM and IMM+TRN groups before and after the 21-day immobilization; circumference, reactive hyperemic response and maximum voluntary contraction (MVC) of the grip in day 1, and recovery time constant of muscle oxygen consumption (TcVO_2_mus) and endurance performance in day 2. The age (mean ± SD), height (mean ± SD) and weight (mean ± SD) of the subjects were 23.2 ± 3.3 years, 173.0 ± 7.9 cm and 64.9 ± 11.0 kg in IMM group and 22.3 ± 4.8 years, 173.1 ± 5.1 cm and 66.8 ± 10.5 kg in IMM+TRN group, respectively.

### NIRS device

The NIRS (OMRON, HEO 200) was used to measure oxygenation in biological tissue *in vivo *by utilizing the absorption characteristics of oxygenated and deoxygenated hemoglobin (Hb) and myoglobin (Mb). The NIRS device projects light from the near-infrared wavelength region, the region between visible and infrared light, into the body. The device is composed of probe and main computer body, and the probe is equipped with emitter and detectors for near-infrared light of 760 nm and 840 nm wave lengths. The emitted light passes through the skin and, while scattering, reaches tissue where a portion is absorbed by Hb and Mb and then returns to the detectors. The amount of light that then returns is measured to give approximately the oxygenation and de-oxygenation states of Hb and Mb based on the Beer-Lambert Law [13]. Changes in oxygenated Hb and Mb (oxy-Hb), deoxygenated Hb and Mb (deoxyHb) and total Hb were calculated by the formula that has been established in a previous study [23]. The mean penetration depth of NIRS in living tissue is approximately one-half of the distance between the emitter and detector as verified directly and by Monte Carlo simulation [24]. The distance between the emitter and the detector was set at 30 mm, and the penetration depth was approximately 15 mm from the skin surface.

### Reactive Hyperemia

Reactive hyperemia was carried out in the following fashion: one minute of rest, 5 minutes of cuff occlusion at 300 mmHg, 5 minutes of recovery. We monitored recovery of oxy-Hb and totalHb by using NIRS placed in the center of the muscle belly of the flexor digitorum muscles. The muscle was identified by the isometric contraction. We analyzed the peak value of oxy-Hb in the recovery phase and the time from the end point of occlusion to oxy-Hb peak value. All the values were expressed relative to the overall change from rest to the minimum level during cuff occlusion. As for totalHb, we calculated the peak value of the recovery phase with actual survey value (OD value), and the time from the end point of occlusion to totalHb peak value.

### Recovery Time Constant (Tc) for Muscle Oxygen Consumption (TcVO_2_mus)

Subjects were seated and after measuring MVC the NIRS probe was placed in the center of the muscle belly of the flexor digitorum muscles and the sphygmomanometer cuff was placed around the upper arm. With the subject in a resting condition, measurements were thus begun. VO_2_mus measurement was carried out in the following fashion: one minute of rest, 5 minutes of cuff occlusion at 300 mmHg, 5 minutes of recovery after occlusion, one minute of dynamic hand grip exercise by a hand grip ergometer [25] equipped with a weight-loaded system at 1 repetition per 4 seconds (1 second contraction, 3 seconds relaxation) at 40% MVC as measured before immobilization. Brief arterial occlusions were repeated following the completion of exercise, and VO_2_mus was obtained [11]. The occlusion of arterial blood flow was terminated soon after total amount of Hb reached an almost uniform, constant level. It has been shown in previous research that the percentage of de-oxygenated Hb/Mb at the time of arterial occlusion is a direct index of VO_2_mus [25], and this value of VO_2_mus is shown as fraction of resting value. TcVO_2_mus was calculated from the VO_2_mus value according to the formula shown below and fit to mono-exponential curve:

y = a(1-e^-kt^)

For this equation, *y *represents the relative value of VO_2_mus during arterial occlusion in the rest period following exercise, *a *represents the total amount of change in VO_2_mus from the value at the completion of exercise to the value at recovery, k is the rate constant (1/k = Tc), and t is time.

### Forearm Circumference

Forearm circumference was measured at the point of greatest circumference in the region of the forearm. The site of the initial measurement was marked, and care was taken to measure at the same site following immobilization.

### Maximum Voluntary Contraction (MVC)

MVC was measured using analogue grip dinamometer (TAKEI KIKI KOGYO, Japan) before repeated exercise tests were performed. Three measurements were made, and the largest was used as MVC value.

### Grip Exercise Endurance 

Grip exercise endurance was measured after the completion of NIRS measurement and sufficient period of rest. Following skeletal muscle recovery, dynamic hand grip exercise was carried out at 30% MVC, 1 repetition per second (0.5 second contraction, 0.5 second relaxation), until the point of exhaustion. The time to exhaustion was recorded as the endurance measurement.

### Training

Endurance and strength handgrip training were carried out for the subjects of the IMM+TRN group twice a week. First, intermittent isometric hand grip exercise at 70% MVC for 2 seconds with a 2 second rest interval was repeated 10 times. Next, dynamic handgrip exercise was performed at 30% MVC at a rate of 1 repetition per 1 second until exhaustion.

### Data Analysis

A repeated measures ANOVA (SPSS version 11.5) was conducted to determine differences between IMM and IMM+TRN groups. All values are reported means ± SD. Standard of significance was established as an uncertainty of less than 5% (p < 0.05) and an uncertainty of less than 1% (p < 0.01), respectively.

## Results

**Figure 1:**
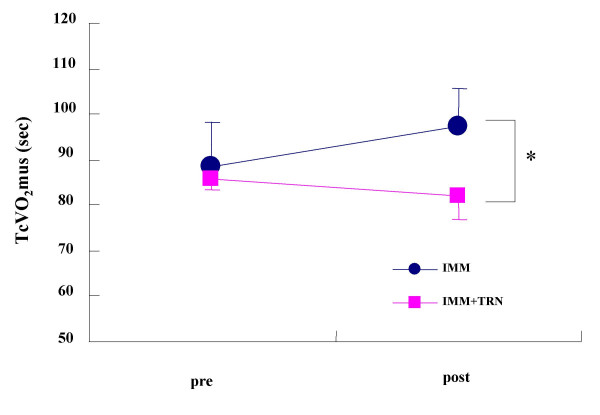
VO_2_mus time constant of the IMM and IMM+TRN groups from pre to post. *Significant group × time interaction was found (p = 0.032, F = 6.711). Values are mean ± SD.

**Figure 2:**
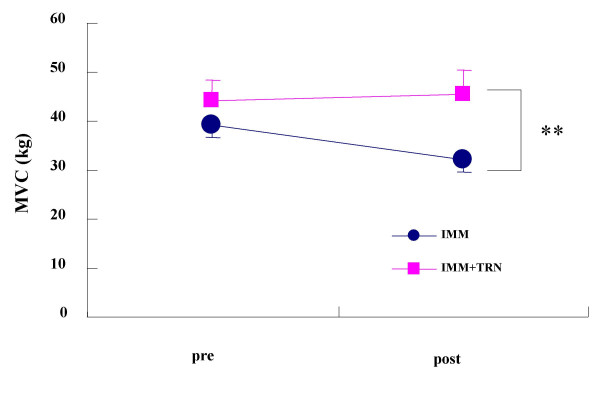
Grip strength of the IMM and IMM+TRN groups from pre to post. **Significant group × time interaction was found (p = 0.001, F = 30.415). Values are mean ± SD.

**Figure 3:**
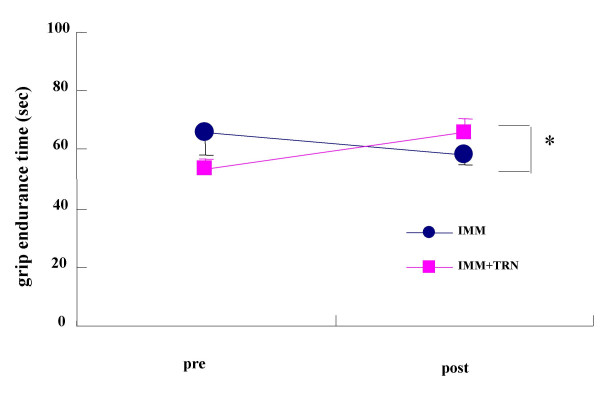
Grip endurance of the IMM and IMM+TRN groups from pre to post. *Significant group × time interaction was found (p = 0.014, F = 9.791). Values are mean ± SD.

For all the measurements made before immobilization, there was no significant difference observed between IMM and IMM+TRN groups. The results of forearm circumference and reactive hyperemia response for the IMM group and the IMM+TRN group before and after immobilization are as shown in Table 1. No significant group-by-time interaction was seen in forearm circumference and reactive hyperemic oxygenation response either in IMM or IMM+TRN group. Figure 1 demonstrates a significant group-by-time interaction (p = 0.032, F = 6.711) between the IMM and IMM+TRN groups in the TcVO_2_mus. A significant group-by-time interaction (p = 0.001, F = 30.415) was observed between the IMM and IMM+TRN groups in the MVC (Figure 2). A significant group-by-time interaction (p = 0.014, F = 9.791) was seen between the IMM and IMM+TRN groups in grip endurance (Figure 3).

**Table 1:** Results of measurements of forearm circumference and reactive hyperemic oxygenation response of the IMM and IMM + TRN groups before and after immobilization.

	Group	Pre	Post
Circumference (cm)	IMM	24.7 ± 1.6	24.7 ± 1.6
	IMM+TRN	24.8 ± 1.4	25.1 ± 1.7
Oxy-Hb peak value (%)	IMM	161.3 ± 17.0	149.6 ± 13.9
	IMM+TRN	149.8 ± 9.5	135.6 ± 18.7
Oxy-Hb ΔT (sec)	IMM	64.3 ± 38.7	75.3 ± 28.1
	IMM+TRN	55.1 ± 22.9	83.5 ± 27.0
Total Hb peak value (%)	IMM	0.072 ± 0.014	0.060 ± 0.019
	IMM+TRN	0.084 ± 0.007	0.063 ± 0.038
Total Hb ΔT (sec)	IMM	45.3 ± 20.2	55.6 ± 38.1
	IMM+TRN	31.4 ± 15.2	48.7 ± 21.7

oxy-Hb peak value, the peak value of oxygenated hemoglobin in the recovery phase after ischemia; oxy-Hb ΔT, the time from the end point of ischemia to oxy-Hb peak value; totalHb peak value, the peak value of total hemoglobin in the recovery phase after ischemia; totalHb ΔT, the time from the end point of ischemia to totalHb peak value. No significant group × time interaction was seen in the forearm circumference and reactive hyperemic oxygenation response either in IMM or IMM+TRN group. Values are mean ± SD.

## Discussion

We found decreases in muscle functions such as grip strength, muscle endurance and oxidative capacity after 21-day upper limb immobilization for the IMM group. However, no change was found in forearm circumference in the IMM group. For the IMM+TRN group, it was found that short duration of exercise training (about 2 min per training) for endurance and strength prevented immobilization-induced decreases in muscle functions.

### Muscle Morphology

In contrast to the present study utilizing upper limb immobilization, a large number of previous studies found decreases in the thigh [26,27] and the calf [28,29] muscle cross-sectional area (CSA) with lower limb immobilization (12% to 21% decrease for 10-day to 4-week immobilization, 8% to 23% for 2-week to 6-week immobilization, respectively). Although there is limited information available regarding upper limb immobilization, it seems that forearm immobilization does not induce a decrease in muscle CSA as evaluated both by magnetic resonance imaging (MRI) and circumference measurement during rather short duration (3 weeks) of immobilization [10]. In this study, we used forearm circumference, an indirect parameter, in attempting to evaluate muscle CSA. In light of the result obtained by Kitahara et al.[10], which utilized the same immobilization protocol, it was hypothesized that there would be no change in forearm CSA after 3-week immobilization in this study as well. The lack of muscle atrophy observed may be partly due to the fact that the upper limb has lower muscle mass than the lower limb and does not possess anti-gravity function. However, since a previous study [30] found a 5% decrease in forearm CSA after 5-week immobilization, it may also be the case that the 3-week immobilization period used in this study may not be sufficiently long to induce muscle morphological change.

### Muscle Function

We found the decreases in both grip strength (-18.2%, p < 0.01) and muscle oxidative capacity (-10.6%, p < 0.05) to be present in the IMM group without any morphological changes. This is in agreement with the data obtained in the previous study [11], which showed no change in circumference, -15% in grip strength and -18% in the oxidative capacity. This dissociation between the loss of muscle strength and muscle size has been found in a different study [31] as well (-4.1% decrease in forearm CSA vs. -29.3% decrease in wrist flexion strength).

It is speculated that the immobilization (3 to 5 week) influences muscle strength to a greater degree that muscle mass. There are some reports indicating that the loss of muscle strength resulting from muscle unloading is primarily due to reduced neural activation of myofibers [32,33], given that the decrements in muscle strength after lower limb unloading interventions were not associated with significant muscle morphological changes [34]. It has been reported that muscle recruitment during MVC monitored by MRI in relatively untrained people is lower than that of the trained, indicating a lower activation of motoneurons in subjects with lower muscle function [35]. Thus, the decrease in grip strength without any change in CSA may be partly due to the decrease in muscle recruitment or reduced numbers of the fibers activated per motoneuron. Further study of muscle recruitment pattern using MRI and EMG would be needed for future research.

In this study, we evaluated muscle oxidative capacity by measuring TcVO_2_mus, a method that has been confirmed to be reliable. In a previous study, a correlation was found between the post-recovery TcPCr measured by MRS, a parameter of muscle oxidative enzyme capacity [36], and the recovery time constant for muscle oxygen consumption (TcVO_2_mus) following the completion of handgrip exercise as measured by NIRS [37]. The prolongation of TcVO_2_mus found in this study is in agreement with the data obtained by Kitahara et al. [10]. The reduction of oxidative capacity may be due to the decrease in mitochondrial enzyme activity [38] and the decrease in selective type 1 fiber area [39].

### Training

In a previous study, the endurance training at 30% of MVC, one contraction per second until exhaustion, 5 times a week (about 60 seconds a day) was reported to increase endurance performance by 39% and oxidative capacity by 30% in healthy women [40]. The same endurance training, but with reduced training frequency to twice weekly, was effective to prevent the immobilization-induced decrease in endurance performance and oxidative capacity, but not to prevent the MVC decrease [11]. In this study, we added the strength training to the endurance training program with an interval in between and successfully prevented the decrease in muscle functions. Generally, strength training comprises an intensity from 60% to 90% of MVC, endurable repetitions (10 to 15 times), and 2 to 3 days weekly [41]. In this study, we used a lower volume program comprised of intermittent isometric (2 seconds contraction, 2 seconds relaxation) grip exercise at 70% of MVC, 10 repetitions, and twice weekly so that the subjects could perform the training without any difficulties. This low volume training program was effective to preserve grip strength even after 3-week immobilization (-18.2% for IMM and 2.6% for IMM-TRN groups). However, a variety of training programs should be tested to determine the minimum training volume for preserving grip functions after immobilization. An exercise program focused on the grip functions would likely serve as a relevant measure to preserve forearm function, specifically for astronauts working outside a spacecraft [42], the elderly (showing 3 to 5% decrease in grip strength per year) [43], and long-term bed-ridden patients.

### Reactive Hyperemia

Reactive hyperemia has been evaluated using plethysmography and ultrasonography [44]. The increase in shear stress resulted from the abrupt increase in blood inflow after liberation of ischemia facilitates endothelial nitric oxide (NO) production to induce vasodilatation. It is believed that NO plays a major role for arterial vasodilatation after ischemia applied with a duration of less than 5 minutes.

We hypothesized that 3-week forearm immobilization would reduce endothelial NO production resulting in the attenuation of reactive hyperemia, and that exercise training may partially reverse the blunted endothelial function. We also anticipated that data obtained by NIRS may provide similar measures to those of plethysmography and ultrasonography. We used NIRS to measure for oxy-Hb and totalHb the times to reach peak hyperemia and the peak values after ischemia. However, neither of the indicators showed any significant change between pre- and post-immobilization and between IMM and IMM+TRN groups.

Reactive hyperemic blood flow in the superficial femoral artery was significantly decreased by 28% after 52-day bed rest experiment [3], and tended to be lower after 4-week leg immobilization [4] in healthy volunteers. Hyperemic response is also reduced for patients with diabetes [45], renal failure [46]. These results indicate that the vasodilatation capacity for resistance vascular bed was reduced by whole body immobilization. Some plausible explanations for not observing any significant changes in post-ischemic NIRS indicators after 3-week immobilization in this study are the selection of the subjects (young healthy volunteers) and the short immobilization duration. Since the subjects were instructed to maintain daily physical activity level, this activity might counter the decrease in endothelial function. It is also reported that grip exercise training partially reversed attenuated reactive hyperemia in patients with chronic heart failure [47]. However, moderate exercise training was not found effective for sedentary people [48]. Also, although highly trained professional tennis players showed higher endothelial function in the dominant arm than in the non-dominant arm [49], no difference was observed between dominant and non-dominant arms in non-athletic counterparts. These results indicate that a drastic stimulus of exercise stress to the vascular bed is needed to enhance endothelial function. To examine the effects of grip exercise training of this study on the endothelial function, we should select individuals with distorted endothelial function, such as patients with cardiovascular risk factors, and also consider the physical activity level of the subjects in addition to the training protocol for grip exercise.

## Limitation

Subcutaneous adipose tissue thickness has a substantial confounding influence on muscle NIRS measurements. The influence of adipose tissue thickness on the NIR spectra of human muscle was studied by Monte Carlo simulations of a two-layer structure and with phantom experiments. The study suggested that subject-to-subject variation in the fat thickness can be ignored if the fat thickness is less than 5 mm. Other studies indicated that for a fat thickness of 5 mm the signal intensity reduces approximately by 0.2 with a light source-detector separation being 30 to 40 mm. We have not measured the skin-fold thickness of the subjects in this study. But it should be less than 2 mm with less variability according to the age and BMI and subcutaneous adipose tissue thickness should not be changed after immobilization [10]. So we believe that subcutaneous adipose tissue thickness did not influence the results of this study.

## Conclusion

We observed a decrease in muscle functions after 3-week forearm immobilization. However, the low volume exercise training (about 2 minutes per training) for endurance and strength reduced the magnitude of decreases in muscle functions induced by immobilization. This low volume training may be suitable to clinical application also on old subjects. We were unable to find any changes in post-ischemic NIRS indicators after 3-week immobilization.

## Authors' contributions

MM performed selection and medical checks of the experiment subjects as well as the measurement and evaluation of NIRS data. TH served as the general administrator for the experiment and performed the cast immobilization process. NM and TO performed selection and medical checks of the experiment subjects. YK and KE performed measurement and evaluation of NIRS data. CU, SI, KS, FO and KY also performed measurement and evaluation of NIRS data. TK and AH examined the training method. All authors read and approved the final manuscript.
